# A realist evaluation to identify contexts and mechanisms that enabled and hindered implementation and had an effect on sustainability of a lean intervention in pediatric healthcare

**DOI:** 10.1186/s12913-019-4744-3

**Published:** 2019-11-29

**Authors:** Rachel Flynn, Thomas Rotter, Dawn Hartfield, Amanda S. Newton, Shannon D. Scott

**Affiliations:** 1 Faculty of Nursing, Level 3, Edmonton Clinic Health Academy, University of Alberta, 11405 87 Avenue, Edmonton, Alberta T6G 1C9 Canada; 2 Healthcare Quality Programs, Queen’s University School of Nursing, Kingston, Ontario K7L 3N6 Canada; 3 Department of Pediatrics, Faculty of Medicine and Dentistry, University of Alberta, 11405 87 Avenue, Edmonton, AB T6G 1C9 Canada

**Keywords:** Sustainability, Normalization, Lean, Quality improvement, Realist evaluation

## Abstract

**Background:**

In 2012, the Saskatchewan Ministry for Health mandated a system-wide Lean transformation. Research has been conducted on the implementation processes of this system-wide Lean implementation. However, no research has been done on the sustainability of these Lean efforts. We conducted a realist evaluation on the sustainability of Lean in pediatric healthcare. We used the context (C) + mechanism (M) = outcome (O) configurations (CMOcs) heuristic to explain under what contexts, for whom, how and why Lean efforts are sustained or not sustained in pediatric healthcare.

**Methods:**

We employed a case study research design. Guided by a realist evaluation framework, we conducted qualitative realist interviews with various stakeholder groups across four pediatric hospital units ‘cases’ at one acute hospital. Interview data was analyzed using an integrated approach of CMOc categorization coding, CMOc connecting and pattern matching.

**Results:**

We conducted thirty-two interviews across the four cases. Five CMOcs emerged from our realist interview data. These configurations illustrated a ‘ripple-effect’ from implementation outcomes to contexts for sustainability. Sense-making and staff engagement were prominent mechanisms to the sustainment of Lean efforts. Failure to trigger these mechanisms resulted in resistance. The implementation approach used influenced mechanisms and outcomes for sustainability, more so than Lean itself. Specifically, the language, messaging and training approaches used triggered mechanisms of innovation fatigue, poor ‘sense-making’ and a lack of engagement for frontline staff. The mandated, top-down, externally led nature of implementation and lack of customization to context served as potential pitfalls. Overall, there was variation between leadership and frontline staff’s perceptions on how embedded Lean was in their contexts, and the degree to which participants supported Lean sustainability.

**Conclusions:**

This research illuminates important contextual factors and mechanisms to the process of Lean sustainment that can be applicable to those implementing systems changes. Future work is needed to continue to develop the science on the sustainability of interventions for healthcare improvement.

## Background

Lean is a quality management system aimed at maximising value for customers by reducing waste (e.g., overproduction, wait times, unnecessary inventory and motion) and reconfiguring organizational processes [1–4]. Lean is increasingly recognized as a potential approach to improve problems in healthcare [5]. Lean has been implemented in a variety of healthcare settings (e.g., emergency departments, outpatient clinics, pediatric care) [6–9], through a variety of implementation approaches (e.g., mandated at macro level, driven by frontline staff at meso level of a system), targeting various levels of healthcare systems (macro, meso or micro). It has also been conceptualized in multiple ways (e.g., a philosophy or management system versus quality improvement (QI) tools) [10–13].

The majority of previous research on Lean implementation in healthcare has not addressed the contextual factors and mechanisms that influence the sustainability of Lean efforts [14–18]. Understanding these factors that contribute to its lasting effect are as important as understanding how to implement Lean in the first place [19]. Sustainability is an important yet understudied area of implementation research [20, 21]. This paper presents the final phase of a multi-phase realist investigation on the sustainability of Lean efforts in pediatric healthcare. Phase 1 consisted of initial program theory development. Phase 2 consisted of a realist review to further develop and refine our initial program theory [22]. Phase 3, reported in this paper, was a realist evaluation to test and refine our program theory and context (C) + mechanism (M) = outcome (O) configurations (CMOcs) developed during phase 1 and 2 of this research.

### Research aim

Our research aim was to generate, test and refine a program theory on the sustainability of Lean efforts in pediatric healthcare using a realist approach. We drew from the conceptualization that sustainability is the continuation or the integration of new practice within an organization whereby it has become a routine part of care delivery and continues to deliver desired outcomes, whereby the ways of thinking and attitudes behind processes and outcomes have changed and the new practice has become the new way of working [23]. For our research we undertook a realist approach to identify the contextual factors and mechanisms that influenced whether Lean became a routine part of care delivery, continued to deliver desired outcomes and became the sustained, normalized way of working. We aimed to identify participants’ perceptions of Lean implementation processes, contextual factors, resources and mechanisms that enabled or hindered the sustainment of Lean efforts.

## Methods

### Study design and setting

We followed a realist evaluation methodology framework [24] with an explanatory case study research design [25]. We defined a ‘case’ as a pediatric unit involved in Lean implementation from one acute hospital setting in the Saskatchewan health system. One central tenet of realist methodology is that programs work differently in different contexts [24]; hence, we chose to conduct interviews across various cases (units) where Lean implementation had occurred in the research context of the Saskatchewan health system.

In 2012, the Saskatchewan Ministry of Health committed a multi-million dollar investment to implement a broader system-wide Lean transformation, led by an external consulting group [26]. This has been titled “*the largest Lean transformation in the world”* [27]. The overarching aim was to create “better health, better value, better care, and better teams” [28]. Early-stages of this implementation focused on leadership training, and the creation of Kaizen Promotion Offices (KPOs) to provide supportive infrastructure for the Lean transformation [29]. This was intended to build internal capacity and capability for continuous quality improvement [30]. The Saskatchewan Lean management system was used in combination with Hoshin Kanri, and daily visual management [31] and used a variety of Lean tools and activities (e.g., Rapid Process Improvement Workshops (RPIW’s), Mistake Proofing, Kanban, and Value Stream Map). The Saskatchewan Ministry of Health [28] proposed that, “Lean empowers employees to find ways to improve. It focuses on identifying and reducing waste. In healthcare, that would include things like excess inventory, time spent waiting for services, and inefficient processes that reduce time spent on direct patient care.”

This large-scale Lean transformation created a novel opportunity for our research on the sustainability of Lean across multiple pediatric healthcare settings. There is no prior evaluation on Lean sustainability in Saskatchewan and none specific to pediatric healthcare. Pediatric healthcare was an important context for us to study under the Saskatchewan Lean management system due to the development of the provinces first children’s and maternal hospital based on Lean.

### Ethics approval

Ethics approval for this study was granted by the University of Alberta Health Research Ethics Board and the University of Saskatchewan Behavioural Research Ethics Board. Institutional approval was provided by the Saskatoon Health Region, Inter-professional Practice, Education and Research office.

### Recruitment and data collection

To develop an understanding of how Lean efforts were embedded in practice, we purposefully selected four pediatric units (a pediatric inpatient unit, outpatient unit, pediatric intensive care unit (PICU) and neonatal intensive care unit (NICU) involved in the effort. All staff from each included case were invited to participate in the interviews to gain broad and diverse perspectives. Staff was invited to participate through communication by the unit managers at each case and the Director of children’s services. A range of perspectives via professional roles were purposefully sought to contribute to refinement of the program theory.

We conducted qualitative realist interviews [32] using an interview guide (Additional file 1.) to test and further refine our initial program theory (developed in phase 1), the CMOcs substantiated in our realist review (phase 2) and to explore new emerging relevant CMOcs. The interviews explored participants’ perceptions of Lean, implementation processes, contextual factors, resources and mechanisms that enabled or hindered the sustainment of Lean efforts. Interviews were conducted using semi-structured interview guides. All interviews were conducted in person or by telephone by the lead author (RF), audio recorded and transcribed. Realist interviews explicitly discuss the program theory with the participants, giving them the opportunity to confirm, refute or refine the theory, this is described as the teacher-learner cycle [24, 32].

### Methodological approach: realist evaluation

A realist approach offers ways to address how, when, why and where the intervention works or not through the generation of an explanatory program theory [33]. Realist evaluations are driven by the question: what works, how, for whom, in what circumstances and to what extent? [24]. A realist evaluation is underpinned by the context (C) + mechanism (M) = outcome (O) configurations (CMOcs) heuristic [24]. A CMOc is a hypothesis that the program works (O) because of the action of some underlying mechanism (M), which only comes into operation in particular contexts (C) [34]. The development, testing and refinement of CMOcs in a realist evaluation provides causal explanation of how and why a program works [35]. The realist terms used for this research are detailed in Table 1.

**Table 1:** Realist terminology

Terminology	Explanation
Context-mechanism-outcome configurations (CMOc)	“CMO configuring is a heuristic used to generate causative explanations about outcomes in the observed data. A CMO configuration may be about the whole program or only to certain aspects. One CMO may be embedded in another or configured in a series (‘ripple effect’ in which the outcome of one CMO becomes the context for the next in the chain of implementation steps). Configuring CMOs is a basis for generating and/ or refining the theory that becomes the final product of the review” [36].
Context	“Context can be defined as all factors that are not part of the program or intervention itself, the “backdrop” to implementation, yet does interact, influence, modify, facilitate or hinder the intervention and its effectiveness (in our case the sustainability of Lean efforts)” [37].
Mechanisms	Mechanisms are the combination of resources (intended and unintended) offered by a social program under study (Lean) and the response to those resources (cognitive, emotional, motivational reasoning etc.) by stakeholders [24]. “Causal mechanisms are underlying entities, process or structures which operate in particular contexts to generate outcomes of interest” [38].
Outcomes	“Outcomes are a result of a program firing multiple mechanisms which have different effects on different subjects in different situations, and so produce multiple outcomes. Realist evaluators examine outcome patterns in a theory testing role. Outcomes are analyzed to discover if conjectured mechanism/context theories are confirmed” ([24], p., 217).

### Theoretical guidance

We drew upon the National Health Sustainability Model (NHS SM) and Normalization Process Theory (NPT) to help us to understand the contextual factors and mechanisms that trigger the sustainability or otherwise of Lean efforts. The NHS SM identifies ten key contextual factors that increase the likelihood of sustainability and continuous improvement. These factors are grouped into three domains: Process, staff and organization [23, 39]. The NPT is a middle range theory used to understand the implementation, embedding and integration of evidence-based innovations into healthcare settings as a result of people working individually and collectively to enact them [40, 41]. This middle-range theory is concerned with the social organization of the work (implementation), of making practices routine to everyday (embedding), and of sustaining embedded practices in their social contexts (integration) [40]. The key theoretical constructs to NPT are: coherence, cognitive participation, collective action and reflexive monitoring [40]. These constructs of NPT offer potential mechanisms that promote or inhibit the embedding of complex interventions into routine everyday practice and the likelihood of sustainability.

### Data analysis

Interviews were analysed using CMOc heuristic [24]. We followed Maxwell’s [42] categorising and connecting strategies for data analysis. Firstly we analysed each case separately as a ‘whole study’ and then analysed and summarized similar and/or opposing evidence across the four included cases through data triangulation and pattern matching [25]. During categorization, C, M, O coding for each case was done by a process of data extraction using a bespoke form and coding using NVivo 11 software. Cross case comparisons were made to determine how the same causal mechanisms played out in different contexts and produced the same or different outcomes. Connecting data involved pattern matching across cases and CMOcs and higher abstraction. This was done using Microsoft Word 13 and Microsoft Excel 13. During the process of analysis it became clear that significant CMOcs were not confined to the cases which we had identified as contexts but rather by contextual elements (e.g., work areas that experience constant change) that triggered mechanisms (e.g., staff felt overwhelmed from the constant change) that lead to outcomes (e.g., negative perceptions of Lean, resistance and lack of support by frontline staff). Thus we analyzed these CMOc patterns within and across cases.

A visual model was developed to show CMOcs patterns across cases (Additional file 2.). These patterns denote the causal pathways leading to program outcomes. Building upon our previous realist review we showed how CMOcs can be linked to each other - with some outcomes of early implementation becoming an aspect of context for sustainability, this is known as the ‘ripple effect’ [36].

Analysis was iterative, going ‘back-and-forth’ between the program theory and the CMOcs from phase 2 and the data gathered in phase 3. The intent was to deliberately attempt to refine and specify the program theory on the sustainability of Lean in pediatric healthcare. We also drew upon our middle-range theories to explain contextual factors and causal mechanisms for the sustainability of Lean in the research context under study. The NHS SM was used to explain process, staff and organization factors that influenced the sustainability of Lean from the experiences of participants. NPT was used to identify any mechanisms related to coherence, cognitive participation, collective action and reflexive monitoring that either hindered or facilitated the embedding of Lean from the perceptions of the participants.

### Validity

Under a realist lens, the focus of validity is the judgement of the degree to which the researcher has encapsulated the multiple perspectives pertaining to a given situation ([43], p82). We followed Pawson et al. [44] Transparency, Accuracy, Purposivity, Utility, Propriety, Accessibility and Specificity (TAPUPAS) criteria to enhance the trustworthiness of data collection and documentation. The steps we took to meet the TAPUPAS criteria are outlined in Table 2.

**Table 2:** TAPUPAS Quality standards framework

TAPUPAS	Quality standards description	Link to phase 3 of research
Transparency	“The process of knowledge generation should be open to outside scrutiny. For knowledge to meet this standard, it should make plain how it was generated, clarifying aims, objectives and all the steps of the subsequent argument, so giving readers access to a common understanding of the underlying reasoning” ([44], p., 38).	We have discussed our aims, theoretical guidance, setting, methods and process of data analysis.
Accuracy	“All knowledge claims should be supported by and faithful to the events, experiences, informants and sources used in their production. For knowledge to meet this standard, it should demonstrate that all assertions, conclusions and recommendations are based upon relevant and appropriate information” ([44], p., 38).	We used participant’s quotations to accurately report the perspectives gathered and show how these perspectives informed the CMOcs identified during analysis.
Purposivity	“The approaches and methods used to gain knowledge should be appropriate to the task in hand, or ‘fit for purpose’. For knowledge to meet this standard, it should demonstrate that the inquiry has followed the opposite approach to meet the stated objectives of the exercise” ([44], p., 38).	We identified that a realist evaluation of multiple stakeholders across multiple cases experiencing the program in question would enable us to explore the CMOcs identified during the realist review. We conducted triangulation using date from a realist review and evaluation to address our research question. We also used middle-range theory during each of those research phases.
Utility	“Knowledge should be appropriate to the decision setting in which it is intended to be used, and to the information need expressed by the seeker after knowledge. For knowledge to meet this standard, it should be ‘fit for use’, providing answers that are as closely matched as possible to the question” ([44], p., 39).	We gathered multiple perspectives of multiple stakeholder professions across multiple cases in the decision setting studied. We also demonstrate limitations to data collection and other sources of knowledge that would have added to utility.
Propriety	“Knowledge should be created and managed legally, ethically and with due care to all relevant stakeholders. For knowledge to meet this standard, it should present adequate evidence, appropriate to each point of contact, of the informed consent of relevant stakeholders. The release (or withholding) of information should also be subject to agreement” ([44], p., 39).	We followed ethical procedures of informed consent for all participants and the ethical guidelines of the research boards that granted ethical approval. Each participant read and signed informed consent before each interview. Data was audio recorded, transcribed and anonymized.
Accessibility	“Knowledge should be presented in a way that meets the needs of the knowledge seeker. To meet this standard, no potential user should be excluded because of the presentational style employed” ([44], p., 40).	This reporting uses academic language for journal publication standards. This research will also be fed back to the organization in the form of an evidence brief form and lay language summary presentation.
Specificity	“The knowledge must pass muster within its own source domain, as perceived by its participants and proponents” ([44], p., 40).	We followed RAMSES II reporting standards for realist evaluations [45].

## Results

### Participant demographics and lean training

Thirty-two realist interviews were conducted; nine participants from case 1, three participants from case 2, seven participants from case 3 and four participants from case 4, and nine participants that were across cases. The total sample consisted of registered nurses (*n* = 9), unit managers (*n* = 5), physicians (*n* = 4), allied health professionals (*n* = 4), clinical nurse educators (*n* = 2), care assistants (*n* = 2), senior leaders (*n* = 4), and parents (*n* = 2). Of the thirty-two participants, 30 were female and two were male. The majority of participants had been working in their profession from 1 to 5 years (*n* = 9), 31–35 years (*n* = 6), 6–10 years (*n* = 4) or 16–20 years (*n* = 4). The remaining participants had been working in the profession between 11 and 15 years (*n* = 3), 21–25 years (*n* = 2), 26–30 years (*n* = 2) or 36–40 years (*n* = 2). The majority of participants had been working in their current role 1–5 years (*n* = 14), or 6–10 years (*n* = 9).

Seventy-five per cent of the participants had received the Kaizen basic Lean training offered through the organization. The most prevalent Lean activities were visual daily management huddles (*n* = 22), visibility walls/wall walks (*n* = 21) and 5 s events (*n* = 19). Lean involvement responses ranged from one to 6 years ago, the most common response was approximately 3 years ago (*n* = 10). Twenty-eight participants (88%) were aware of Lean events currently taking place on their unit.

### CMO configurations

Five CMOcs were evident through the realist interviews across each case (Table 3). We have arranged our findings according to these five configurations. We present the most prominent quotes from participants to illustrate each CMOc.

**Table 3:** CMOcs from realist interview findings

CMOc 1: ‘Ripple- effect’: The funded, mandated, top-down, externally led nature of Lean implementation	The early stages of Lean implementation were funded, mandated, and top-down in nature (C), driven by an external consultancy firm that initially focused on training senior leadership (C). Frontline staff did not feel involved in Lean changes, and they felt pressured to adopt Lean (M). The lean language used did not make sense to staff (M). Training failed to demonstrate a connection between Lean and healthcare, this led to misunderstandings and negative perceptions of Lean. There was a resistance to Lean, a lack of support for Lean and potential staff retention issues (O) which had a ‘ripple-effect’ on contexts for sustainability.
CMOc 2: Lack of fit between Lean and healthcare and a lack of customization to context	The complexity and dynamic nature of healthcare and the unique needs of pediatric patients (C), was perceived as incongruent with the nature of Lean. The translation of Lean to patient care did not make sense for many staff and Lean efforts felt impersonal. Lean training failed to make the connection between Lean and healthcare clear for staff (M) and early stages of implementation led by the consultancy company failed to customize Lean to the local context, this triggered pitfalls to the success of Lean, such as feelings of disconnect and negative perceptions of Lean (M), resulting in a resistance and a lack of support for Lean continuation (O).
CMOc 3: Rapidly evolving healthcare contexts overtime – “innovation fatigue”	Lean was implemented in areas that experience constant change (C), early-stages of implementation involved multiple Lean events for training purposes (C), frontline staff felt overwhelmed from the constant change, they were unsure what changes were due to Lean and felt that Lean was the latest fad (M), this led to negative perceptions of Lean, resistance and lack of support by frontline staff (O).
CMOc 4: Process of Lean customization to context- positive and negative effects	The contract of the external consultancy leading Lean implementation ended (C), placing the continuation of Lean on internal senior leaders and unit managers (C). This led to a process of customization of Lean to local context, through a variety of ways (drop Lean language, less Lean activities, greater involvement of frontline staff). This customization of Lean and shift in implementation triggered positive and negative responses for frontline staff, unit managers and senior leaders (M). As a result, only some Lean efforts became embedded. However, there was variation and discrepancy between senior leaders and unit managers compared to frontline staff on perceptions of how embedded Lean efforts are and the degree to how much they support the continuation of Lean (O).
CMOc 5: Shared values and sense-making processes for normalization	The context of early-stages of implementation (C), failed to trigger sense-making processes necessary for staff to understand Lean and potentially engage and begin to embed Lean into their practice (O). Shared values were evident between Lean principles and staff professional values as healthcare providers. However, value congruency without clear sense-making processes resulted in lack of adoption of Lean behaviours as part of normalized frontline practice. Sense-making processes were hindered by a failure of initial Lean training efforts to translate the principles of Lean into the context of healthcare that would resonate with staff (M). Lean language and the lack of staff involvement in Lean changes also hindered sense-making processes and feelings of engagement. This resulted in negative perceptions of Lean, a lack of buy in and support for the continuation of Lean from frontline staff (O).

### ‘Ripple effect’: the funded, mandated, top-down, externally led nature of lean implementation

The majority of the frontline staff participants viewed the top-down, mandated, and externally led Lean implementation negatively. Some staff felt that Lean was a cost cutting measure, a “fashion fad”, something that was pushed on them, where implementation was too quick and did not have a clear purpose. Most of the unit managers also viewed the use of an external consultancy company negatively. Participants were conscious of the estimated costs of the consultancy company’s fees and felt that this money could have been used more appropriately. The consultancy company was viewed as an outsider pushing a message that didn’t connect with healthcare. In contrast, some unit managers and senior leaders did value the top-down, mandated approach used, stating that changes would not have occurred to the same degree without such an approach.

Media had a powerful influence over participant’s perceptions and attitudes towards Lean. The portrayal of Lean in the media was primarily negative, this triggered negative perceptions of Lean by frontline staff. Lean training by the consultancy company did not make staff feel involved in Lean changes. The Lean language used by the consultancy company did not make sense for many participants and initial implementation efforts failed to connect Lean to the context of pediatric healthcare. These factors triggered outcomes of resistance from early-stages of implementation, these implementation outcomes in-turn served as hindering contexts for sustainability. Quotes to support this CMOc are presented in Additional file 3.

### Lack of fit between lean and healthcare and a lack of customization to context

In addition to the externally led, mandated implementation of Lean, there was a lack of fit between Lean principles and the healthcare context (e.g., cars versus patient care). There was also a lack of customization to context during early stages of implementation. This resulted in some negative effects, particularly for frontline staff and their support of Lean continuation. The lack of customization to local context triggered mechanisms of disconnect, lack of coherence and negative perceptions about Lean.

Pediatric healthcare was discussed as a complex field which requires a family-centred and flexible approach to care, which some participants believed did not align with Lean. Despite these contextual issues, there were evident shared values between Lean principles and participants’ professional values, such as patient safety, efficiency and waste reduction. However, Lean principles were primarily viewed as incongruent with healthcare. The training provided failed to translate Lean concepts, principles and their meanings from a manufacturing perspective to a healthcare perspective. This hindered sense-making processes. These contextual issues and subsequent mechanisms influenced the degree of support for Lean continuation. Quotes to support this CMOc are presented in Additional file 3.

### Rapidly evolving healthcare contexts overtime – ‘innovation fatigue’

The constant changes occurring in the work environment led to feelings of confusion and uncertainty about what changes were as a result of Lean implementation or something else, such as the changes occurring in relation to the new children’s hospital development within this context. The degree of constant change also triggered feelings that Lean would not “stick”, and that it was another “make-work” project. Unit managers expressed that staff were overwhelmed and staff engagement in Lean was a challenge. These challenges were also coupled with a lack of follow up regarding the Lean changes implemented. These contextual issues and mechanisms produced negative perceptions and an unawareness of what changes were due to Lean efforts. Quotes to support this CMO configuration are presented in Additional file 3.

### Process of lean customization to context- positive and negative effects

In 2014, Lean implementation by the consultancy company ended and the continuation of Lean was placed on internal senior leaders and unit managers. This led to the process of Lean customization to local context. This process involved removal of the Lean Japanese language and a less stringent implementation of Lean activities and principles. There was a shift to better involve and engage frontline staff to lead Lean changes. Unit managers recognized staff involvement as an important factor for the normalization of Lean in everyday practice. This customization process was viewed as a positive transition by unit managers. It removed Lean elements that did not resonate with staff. Unit managers believed that this would improve staff involvement, engagement and buy-in. Although it was recognized that the word Lean had negative connotations for frontline staff buy-in, the customization of Lean to the local context did not make a difference to how Lean was perceived and supported by frontline staff. In contrast, customization led some participants to believe that Lean was no longer used or embedded in practice. There was a clear discrepancy between stakeholder groups across various levels of the system, in how much they felt Lean had become embedded in their everyday work and the degree to which they supported the continuation of Lean efforts. Quotes to support this CMOc are presented in Additional file 3.

### Shared values and sense-making processes for normalization

The continuation of Lean efforts and the normalization of Lean in every day practice relied on how staff ‘made sense’ of Lean and whether the values of Lean aligned with their own personal and/or professional values. These were core mechanisms to the sustainability of Lean that were important from early stages of Lean implementation. Lean values of efficiency, patient safety, and waste reduction were congruent with participants’ professional values as healthcare providers. However, Lean training failed to translate how the principles of Lean aligned with the context of healthcare. Sense-making by frontline staff was hindered by a) the implementation approach used (top-down, use of an external consultancy firm), b) the Lean training received by the consultancy company and c) the type of Lean messaging by media and the consultancy company. It is important to note that this was not the case for the unit managers, who supported the continuation of Lean. Quotes to support this CMO configuration are presented in Additional file 3.

## Discussion

### Theoretical guidance

The NHS SM served as an appropriate middle-range theory to identify and explain contextual factors that influence the likelihood of Lean sustainability. Factors such as staff involvement and training to sustain the process, staff attitudes towards sustaining the change, credibility and adaptability. NPT served as an appropriate middle-range theory to identify and explain mechanisms of change and provided an explanatory model of the normalization of Lean in everyday practice. As identified processes of individual and communal sense-making (coherence), degree of cognitive participation and collective action influence the degree to which Lean efforts are embedded. A realist stance helped to address the complexity of translating Lean to healthcare or provide explanations of what works, for whom, in what respects, to what extent, in what contexts, and how?

### ‘Ripple-effect’

The ‘ripple-effect’ is based on the idea that a program (Lean) is a series of “*events in the history of a system, leading to the evolution of new structures of interaction and new shared meanings* ([46], p., 267). The ‘ripple-effect’ in our research shows the causal relationship between Lean implementation and sustainability, and how implementation processes and outcomes shape sustainability. Our realist interviews primarily illustrate how implementation outcomes (e.g., resistance, lack of customization to context and negative perceptions), nature of implementation (e.g., training that did not connect the meaning of Lean to healthcare, external Lean consultants that were not from healthcare), and the implementation approach (e.g., mandated top-down approach) shape the contexts (resistance, lack of customization and negative perceptions and variation in Lean training and exposure); mechanisms (e.g., degree of sense-making, staff engagement, awareness); and outcomes (e.g., degree of support, continuation and normalization) for the sustainability of Lean efforts.

Our findings also highlight incongruence between leadership (i.e., senior leaders and unit managers) versus frontline healthcare providers in relation to the degree of normalization and continued support of Lean. Similar to recent research findings by Goodridge et al. [47], our research revealed that major gaps remain in the normalization and sustainment of Lean efforts into everyday practice, particularly among frontline staff. For the purposes of this discussion, we would like to focus on four key points that have influenced the normalization process of Lean in our research findings:

1. The use, approach and effect of an external consultancy company to lead early-stages of implementation.
2. The importance of customization to context.
3. The importance of shared values, sense- making and engagement for normalization.
4. The interface of Lean along the hierarchical structures of healthcare and the resulting incongruence between leadership and frontline staff.

### The use, approach and effect of an external consultancy to lead early-stages of implementation

In our research, the use of an external consultancy company to lead implementation was primarily perceived negatively, as an outsider that did not understand healthcare. Concern about the cost of the consultancy company was also raised. An average of over $19 million Canadian dollars (CAD) in consulting fees were paid for a 2-year term [29] with an average cost of over $46 million CAD for Lean implementation in Saskatchewan between 2012 and 2014 [29]. There is variation about whether top-down large-scale transformations or bottom-up, small-scale incremental improvements are more effective [7, 48–50]. Braithwaite [50] argues that complex systems, such as healthcare, will not change because one mandates a solution. Instead, complex systems adapt overtime to suit their own norms, values, practices and contexts [51]. Our research confirms an adaptation over time but begs the question: how much adaption is acceptable in order to determine if Lean efforts are sustained?

Training and messaging by the consultancy company, as an implementation approach, had negative effects for some participants. The early-stages of implementation focused on senior leadership capacity building, through Lean leadership training. The focus on senior leadership resulted in an unintended negative consequence, that frontline staff did not feel involved and instead felt pressured to adopt Lean. Yet staff engagement is critical to the success of adoption [7, 48]. A recent study on the implementation process of Lean in Saskatchewan [47] found that those with Lean leadership training, were more likely to see potential in the value of Lean and support the use of Lean for their work.

The nature and type of Lean training and participation in Lean activities has implications for the extent of normalization. Though training and resources are important to any implementation of organizational change [26], simply receiving training is not sufficient. Our findings show that the nature and approach of the training and resources used are critical to change. Training needs to involve and engage participants and closely emulate the local environment [52]. The most efficacious training is tailored to context, the target audience and based on evidence and feedback [52–55]. Our findings highlighted that initial training failed to demonstrate a connection between Lean and healthcare which triggered negative perceptions and resistance to Lean. This shows that perhaps it is not the mode of delivery that needs consideration, but the messaging used during training.

In contrast to the above findings, senior leadership noted that without the use of a consultancy company and a mandated top-down implementation approach, changes may not have occurred or occurred at a much slower pace. Contrary to our findings, Fine [56] suggested that Lean engages frontline staff, in the sense that staff develop and make the changes. This poses the question of whether a top-down implementation approach and use of a consultancy company contributed to the lack of staff engagement in our research context. As discussed by Braithwaite [50] people resist change that is imposed by others and that mandated change is never given the same weight as clinically driven change.

### The importance of customization to context

Similar to our realist review findings [22], the degree to which mechanisms occurred was influenced by external pressures to use Lean [57], the complexity of care processes [7], the fit between Lean and local context [7, 57]; and other competing needs or demands [58], such as the constant change in healthcare environments. Early stages of implementation led by the consultancy company failed to customize Lean to local contexts, this triggered some pitfalls to the normalization of Lean in practice (e.g., feelings of disconnect, negative perceptions, resistance to Lean and a lack of support for Lean).

The constant change and “innovation fatigue” experienced by participants was one critical contextual factor. Similar to other findings, this can result in Lean being considered another “fashion fad” or “flavor of the month,” [56] that can lead to potential negative effects on adoption. Complexity was also raised as an important contextual factor, which can affect adoption and normalization [51, 55]. A failure to understand how and why the complexity of context influences the process of normalization will impact the use and sustainability of Lean in healthcare [59–62]. Our findings supplement the existing argument that it cannot be assumed that the translation of Lean from manufacturing to healthcare without consideration of context will offer the same benefits as achieved by Toyota [15, 57].

It is well supported that context is critical to the degree of success in the implementation of large-scale interventions [63–66]. Contextual factors can have a direct effect on the uptake and outcomes of interventions [64, 65]. Complex interventions that struggle to integrate into existing contexts are unlikely to be normalized [55]. It is also important to note differences in terms of macro level (system) contexts. Examples of successful Lean implementations in health systems across America (e.g., Virginia Mason, Seattle Children’s Hospital) may prove different in the context of Canadian healthcare where funding models, insurance models, and governance are different.

Waring and Bishop [18] suggest that Lean is likely to be adapted to ensure it fits with the contexts for clinical practice. The process of customization to existing contexts may facilitate the normalization of interventions [67], such as Lean. In the context of our research study, when the consultancy company contract ended this led to a process of customization to the context. However, despite this shift from overt Lean implementation to implicit implementation, there was still variation to the degree to which people supported the continuation of Lean. There was clear discrepancy between leadership and frontline staff perceptions on how much they supported the continuation of Lean. This poses questions around the process and timing of customization to context, the degree of influence of early-stages of implementation on sustainability and the influence of organizational hierarchical structures on sustainability.

### The importance of shared values, sense- making and engagement for normalization

In addition to a receptive context, Greenhalgh et al. [68] argued there also needs to be a good fit between the program being implemented and the needs and values of the potential adopters. The degree that staff values an intervention or program from early-stages of implementation is associated with the degree of effective adoption [69–71]. In our findings, the nature of the Lean training, poor knowledge translation strategies (e.g., education, training, audit and feedback) and external Lean consultants hindered frontline staff engagement and sense-making. Our realist review found that the more people value the change being implemented the more likely they will engage in the implementation efforts [72]. However, our realist interviews showed that despite shared values with Lean (e.g., patient safety, efficiency, waste reduction), normalization did not occur due to failed sense-making processes from early-stages of implementation. These issues make Lean implementation a highly contested process [18, 73].

To facilitate normalization, it is necessary to appeal to the values and reasoning of potential adopters [73]. Fine et al. [56] argue that those who truly make sense of Lean will see its value for their work and subsequently begin to apply it. This study describes the idea of the “tipping point” where leaders no longer had to “push” Lean ideas out to staff. Instead, staff “pull” Lean and demand it for themselves ([57], p34). It appears this was the intention in Saskatchewan, when there was the shift in the implementation approach. However, reflecting on our findings, it seems the “tipping-point” has not come to fruition yet. Sense-making about Lean may occur during early stages of implementation but is equally as important to maintain for the normalization and sustainability of Lean efforts. Another pitfall in our findings that affected sense-making processes was the ways in which Lean was messaged, the lack of “stickiness” to the Lean messaging used, in other words the lack of natural appeal for frontline staff [74, 75]. The concept of “stickiness” is required for effective messaging and uptake.

Our findings also demonstrated that frontline staff engagement was hindered by poor messaging, lack of sense-making processes and the implementation approach used. Engagement of nurses has already been found to be an issue with regards to Lean implementation in Saskatchewan. In 2014, a survey conducted by the Saskatchewan Union of Nurses [75] found a statistically significant negative effect of Lean on nurse engagement. Physician involvement is also widely addressed as a critical factor to implementation and QI success [76, 77]. Our study had limited physician participation, the reasoning for poor participation is unknown yet mirrors previous work on Lean implementation in Saskatchewan [47]. Future research that solely focuses on physician perspectives on the Saskatchewan Lean management system would be valuable.

Misunderstandings of Lean also creates staff disengagement [78]. Misunderstandings may be triggered from the overuse of ‘Japanese’ Lean language that does not resonate with all health professionals. Several studies have reported that the conceptualization of Lean in healthcare is unclear and varied [1, 79–81] and may be conceptually challenging for staff [16, 65]. Another issue is the blending of several QI methodologies with Lean, without clear definitions. This makes it difficult to differentiate Lean from other approaches and thus it is hard to evaluate what successes or failures are attributed to Lean or not. There needs to be more consistent and standardized conceptualizations of Lean and clearer differentiations between QI approaches in order to distinguish Lean from other QI approaches. This duty should be a collaborative role of research and leaders in healthcare improvement.

### The interface of lean along the hierarchical structures of healthcare

There were ample differences in interview responses between leadership and frontline staff. Frontline staff portrayed more negative perceptions of Lean in comparison to their unit managers or senior leaders. Similarly, a recent survey on Lean implementation processes in Saskatchewan using NPT [47] found that respondents in leadership positions were much more likely to view Lean implementation and outcomes positively. The results of this survey also found wide variation between the perspectives of leaders and frontline staff regarding the NPT constructs of coherence, cognitive participation and reflexive monitoring. This survey illustrated issues around staff familiarity with Lean principles and activities and perspectives that Lean is not currently a part of their work. It appears that part of these issues are a result of the silo and hierarchical nature of healthcare [82, 83].

This idea of the interface of Lean along the hierarchical structures of healthcare and the impact of professional role status along that hierarchy on the success of Lean implementation requires further exploration. These structures and roles in healthcare may impede the ability to achieve alignment from senior leadership to frontline staff [83]. Alignment is the consistency of plans, visions, resources, actions and results to support system-wide goals [83]. Clear accountability structures and integration are needed for system-wide alignment. Previous work has recognized the hierarchical nature of healthcare and professional silos as a barrier to Lean success [84, 85]. However, it remains unclear how to achieve such changes in highly entrenched hierarchical systems.

## Conclusion

Our research demonstrates a ‘ripple-effect’, that is a causal link between implementation and sustainability. Sustainability is hinged on the degree of success at early-stages of implementation. We identified sense-making and engagement as critical mechanisms to sustainability. Sense-making is facilitated or hindered by certain messaging, training and language used during initial stages of implementation. The degree of sense-making and engagement by staff at early-stages of implementation had a ripple-effect on sustainability. The interface of Lean with the hierarchical structures and professional silos of healthcare also play a role to the degree of normalization of Lean. The traditional hierarchical structures and silos in healthcare may impede the ability to achieve alignment from senior leadership to frontline staff and thus hinder the likelihood of embedding Lean in everyday practice. The customization of Lean to context was also critical to the degree of sustainability. Context is known to have a direct effect on the uptake and outcomes of interventions. However there remains knowledge gaps and questions about the timing of and approach to customization and requires further exploration. This research provides practical guiding principles that healthcare leaders may incorporate into planned Lean implementation.

Our research also identified challenges to evaluating sustainability of complex interventions. There is variation in the literature on the conceptualization of sustainability, measurements and outcomes of sustainability. We recognize like others that there is a need for the development and pilot testing of theoretical frameworks and tools to evaluate the sustainability of complex interventions in healthcare. Without such guidance, it is difficult to develop a science on the sustainability of QI efforts and complex interventions in healthcare. Such developments need to make sense and be applicable to those people using them in health systems. Further work using other methods is needed to examine and further test the mechanisms identified in our realist evaluation in other contexts for theory development and to identify predictors of sustainability.

## Supplementary information

**Additional file 1.** Interview Guide.

**Additional file 2.** CMOc visual model.

**Additional file 3.** Interview quotes to support CMOcs.

## Data Availability

The qualitative data supporting this study is not available as participants did not consent to having their data publicly available. As a result, we are not authorized to share the dataset.

## Abbreviations

CMOc: Context + Mechanism = Outcome configuration
KPOs: Kaizen Promotion Offices
NHS SM: National Health Services Sustainability Model
NPT: Normalization Process Theory
QI: Quality improvement

## References

[1] Rotter Thomas, Plishka Christopher, Lawal Adegboyega, Harrison Liz, Sari Nazmi, Goodridge Donna, Flynn Rachel, Chan James, Fiander Michelle, Poksinska Bonnie, Willoughby Keith, Kinsman Leigh (2018). What Is Lean Management in Health Care? Development of an Operational Definition for a Cochrane Systematic Review. Evaluation & the Health Professions.

[2] Womack JP, Jones DT, Roos D (1990). The machine that changed the world: the story of lean production.

[3] Womack JP, Jones DT (2003). Lean thinking: banish the waste and create wealth in your corporation.

[4] Mann D (2010). Creating a lean culture: tools to sustain lean conversions.

[5] Goodridge D, Westhorp G, Rotter T, Dobson R, Bath B (2015). Lean and leadership practices: development of an initial realist program theory. BMC Health Serv Res.

[6] Mazzocato P, Savage C, Brommels M, Aronsson H, Thor J (2010). Lean thinking in healthcare: a realist review of the literature. Qual Saf Health Care.

[7] Mazzocato P, Thor J, Bäckman U (2014). Complexity complicates lean: lessons from seven emergency services. J Health Organ Manag.

[8] Holden RJ (2011). Lean thinking in emergency departments: a critical review. Ann Emerg Med.

[9] Grove AL, Meredith JO, Macintyre M, Angelis J, Neailey K (2010). Lean implementation in primary care health visiting services in National Health Service UK. Qual Saf Health Care.

[10] McCann L, Hassard J, Granter E, Hyde P (2015). Casting the lean spell: the promotion, dilution and erosion of lean management in the NHS. Hum Relat.

[11] Bhasin S, Burcher P (2006). Lean viewed as a philosophy. J Manuf Tech Manag.

[12] Burgess N, Radnor Z (2013). Evaluating lean in healthcare. Int J Health Care Qual Assur.

[13] Smith G, Poteat-Godwin A, Harrison LM, Randolph GD (2012). Applying lean principles and kaizen rapid improvement events in public health practice. J Public Health Manag Pract.

[14] Young TP, McClean SI (2008). A critical look at lean thinking in healthcare. Qual Saf Health Care..

[15] Radnor Z, Osborne SP (2013). Lean: a failed theory for public services. Public Manage Rev.

[16] Andersen H, Røvik KA, Ingebrigtsen T (2014). Lean thinking in hospitals: is there a cure for the absence of evidence? A systematic review of reviews. BMJ Open.

[17] DelliFraine JL, Langabeer JR, Nembhard IM (2010). Assessing the evidence of six sigma and lean in the health care industry. Qual Manag Health Care.

[18] Waring JJ, Bishop S (2010). Lean healthcare: rhetoric, ritual and resistance. Soc Sci Med.

[19] Chambers DA, Glasgow RE, Stange KC (2013). The dynamic sustainability framework: addressing the paradox of sustainment amid ongoing change. Implement Sci.

[20] Wiltsey Stirman S, Kimberly J, Cook N, Calloway A, Castro F, Charns M (2012). The sustainability of new programs and innovations: a review of the empirical literature and recommendations for future research. Implement Sci.

[21] Proctor E, Luke D, Calhoun A (2015). Sustainability of evidence-based healthcare: research agenda, methodological advances, and infrastructure support. Implement Sci.

[22] Flynn R, Newton AS, Rotter T, Hartfield D, Walton S, Fiander M, Scott SD (2018). The sustainability of Lean in pediatric healthcare: a realist review. BMC Syst Rev.

[23] Maher L, Gustafson D, Evans A. NHS Sustainability Model. NHS Institute for Innovation and Improvement; 2010. http://webarchive.nationalarchives.gov.uk/20160805122935/http://www.nhsiq.nhs.uk/media/2757778/nhs_sustainability_model_-_february_2010_1_.pdf. Accessed 31 May 2018.

[24] Pawson R, Tilley N (1997). Realistic evaluation.

[25] Yin RK (2003). Case study research: design and methods.

[26] Mackenzie J, Hall W (2014). “Lean” in Canadian health care: doing less while achieving more.

[27] Kinsman L, Rotter T, Stevenson K (2014). “The largest lean transformation in the world”: the implementation and evaluation of lean in Saskatchewan healthcare. Healthc Q.

[28] Government of Saskatchewan (2013). Ministry of Health: Plan for 2013–14.

[29] Sari N, Rotter T, Goodridge D, Harrison L, Kinsman L (2017). An economic analysis of a system wide lean approach: cost estimations for the implementation of lean in the Saskatchewan healthcare system for 2012-2014. BMC Health Serv Res.

[30] Saskatchewan-Health-Quality-Council (2014). Lean Reform: Saskatchewan Healthcare Adopts Lean Management for Big Benefits.

[31] Rotter T, Kinsman L, Bath B, Goodridge D, Harrison L, Dobson R (2014). A first phase evaluation of Saskatchewan’s lean health care transformation: final report.

[32] Manzano A (2016). The craft of interviewing in realist evaluation. Evaluation.

[33] Salter KL, Kothari A (2014). Using realist evaluation to open the black box of knowledge translation: a state-of-the-art review. Implement Sci.

[34] Pawson R, Manzano-Santaella A (2012). A realist diagnostic workshop. Evaluation.

[35] Punton M, Vogel I, Lloyd R (2016). Reflections from a realist evaluation in progress: scaling ladders and stitching theory. CDI Pract Pap.

[36] Jagosh J, Bush PL, Salsberg J (2015). A realist evaluation of community-based participatory research: partnership synergy, trust building and related ripple effects. BMC Public Health.

[37] Dopson S, Fitzgerald LA, Dopson S, Fitzgerald LA (2005). The active role of context. Knowledge to action? Evidence-based health care in context.

[38] Astbury B, Leeuw F (2010). Unpacking black boxes: mechanisms and theory building in evaluation. Am J Eval.

[39] Doyle C, Howe C, Woodcock T (2013). Making change last: applying the NHS institute for innovation and improvement sustainability model to healthcare improvement. Implement Sci.

[40] May CR, Mair F, Finch T (2009). Development of a theory of implementation and integration: normalization process theory. Implement Sci.

[41] May C, Finch T (2009). Implementing, embedding, and integrating practices: an outline of normalization process theory. Sociology.

[42] Maxwell JA (2012). A realist approach for qualitative research.

[43] Porter S (2007). Validity, trustworthiness and rigour: reasserting realism in qualitative research. J Adv Nurs.

[44] Pawson R, Boaz A, Grayson L, Long A, Barnes C (2003). Types and quality of knowledge in social care.

[45] Wong G, Westhorp G, Manzano A, Greenhalgh J, Jagosh J, Greenhalgh T (2016). RAMESES II reporting standards for realist evaluations. BMC Med.

[46] Hawe P, Shiell A, Riley T (2009). Theorising interventions as events in systems. Am J Community Psychol.

[47] Goodridge D, Rana M, Harrison EL (2018). Assessing the implementation processes of a large-scale, multi-year quality improvement initiative: survey of health care providers. BMC Health Serv Res.

[48] Wong AM, During D, Hartman M, Lappan-Gracon S, Hicks M, Bajwa S (2016). Lean transformation of the eye clinic at the hospital for sick children: challenging an implicit mental model and lessons learned. Healthc Q.

[49] Hung DY (2016). Spreading lean: taking efficiency interventions in health services delivery to scale. Agency for Healthcare Research and Quality.

[50] Braithwaite J (2018). Changing how we think about healthcare improvement. BMJ.

[51] Braithwaite J, Churruca K, Long JC, Ellis LA, Herkes J (2018). When complexity science meets implementation science: a theoretical and empirical analysis of systems change. BMC Med.

[52] Cresswell KM, Bates DW, Sheikh A (2013). Ten key considerations for the successful implementation and adoption of large-scale health information technology. J Am Med Inform Assoc.

[53] Paré G, Sicotte C, Jaana M, Girouard D (2008). Prioritizing the risk factors influencing the success of clinical information system projects. A Delphi study in Canada. Methods Inf Med.

[54] Ash JS, Stavri PZ, Kuperman GJ (2003). A consensus statement on considerations for a successful CPOE implementation. J Am Med Inform Assoc.

[55] Cummings A, Lund S, Campling N, May CR, Richardson A, Myall M (2017). Implementing communication and decision-making interventions directed at goals of care: a theory-led scoping review. BMJ Open.

[56] Fine BA, Golden B, Hannam R, Morra D (2009). Leading lean: a Canadian healthcare leader’s guide. Healthc Q.

[57] Mazzocato P, Holden RJ, Brommels M (2012). How does lean work in emergency care? A case study of a lean-inspired intervention at the Astrid Lindgren Children’s hospital, Stockholm, Sweden. BMC Health Serv Res.

[58] Northway T, Krahn G, Thibault K (2015). Surgical suite to pediatric intensive care unit handover protocol: implementation process and long-term sustainability. J Nurs Care Qual.

[59] Øvretveit J (2011). Understanding the conditions for improvement: research to discover which context influences affect improvement success. BMJ Qual Saf.

[60] Robinson S, Radnor ZJ, Burgess N, Worthington C (2012). SimLean: utilising simulation in the implementation of lean in healthcare. Eur J Oper Res.

[61] Plsek PE, Greenhalgh T (2001). Complexity science: the challenge of complexity in health care. BMJ.

[62] May C, Johnson M, Finch T (2016). Implementation, context and complexity. Implement Sci.

[63] Radnor ZJ, Holweg M, Waring J (2012). Lean in healthcare: the unfilled promise?. Soc Sci Med.

[64] McCormack B, Kitson A, Harvey G, Rycroft-Malone J, Titchen A, Seers K (2002). Getting evidence into practice: the meaning of ‘context’. J Adv Nurs.

[65] Curran JA, Grimshaw JM, Hayden JA, Campbell B (2011). Knowledge translation research: the science of moving research into policy and practice. J Contin Educ Heal Prof.

[66] Walshe K, Freeman T (2002). Effectiveness of quality improvement: learning from evaluations. Qual Saf Health Care.

[67] Lau R, Stevenson F, Ong BN (2016). Achieving change in primary care—causes of the evidence to practice gap: systematic reviews of reviews. Implement Sci.

[68] Greenhalgh T, Robert G, Macfarlane F, Bate P, Kyriakidou O (2004). Diffusion of innovations in service organizations: systematic review and recommendations. Milbank Q.

[69] Fritz Z, Fuld JP (2015). Development of the universal form of treatment options (UFTO) as an alternative to do not attempt cardiopulmonary resuscitation (DNACPR) orders: a cross-disciplinary approach. J Eval Clin Pract.

[70] Brimblecombe C, Crosbie D, Lim WK, Hayes B (2014). The goals of patient care project: implementing a proactive approach to patient-centred decision-making. Intern Med J.

[71] Weiner BJ (2009). A theory of organizational readiness for change. Implement Sci.

[72] Kim CS, Spahlinger DA, Kin JM, Billi JE (2006). Lean health care: what can hospitals learn from a world-class automaker?. J Hosp Med.

[73] Braithwaite J, Runciman WB, Merry AF (2009). Towards safer, better healthcare: harnessing the natural properties of complex sociotechnical systems. Qual Saf Health Care.

[74] Zander U, Kogut B (1995). Knowledge and the speed of the transfer and imitation of organizational capabilities: an empirical test. Organ Sci.

[75] Saskatchewan Union of Nurses (2014). Lean: A Safe and Effective Tool for Registered Nursing?.

[76] Shortell SM, Bennett CL, Byck GR (1998). Assessing the impact of continuous quality improvement on clinical practice: what it will take to accelerate progress. Milbank Q.

[77] Atkinson P (2004). Creating and implementing lean strategies. Manag Serv.

[78] Van Vliet EJ, Bredenhoff E, Sermeus W, Kop LM, Sol JC, Van Harten WH (2011). Exploring the relation between process design and efficiency in high-volume cataract pathways from a lean thinking perspective. Int J Qual Health Care.

[79] Radnor Z (2011). Implementing lean in health care: making the link between the approach, readiness and sustainability. Int J Ind Eng Manag.

[80] Maijala R, Eloranta S, Reunanen T, Ikonen TS (2018). Successful implementation of lean as a managerial principle in health care: a conceptual analysis from systematic literature review. Int J Technol Assess Health Care.

[81] Radnor Z, Walley P, Stephens A, Bucci G (2006). Evaluation of the Lean Approach to Business Management and its Use in the Public Sector (full report). Edinburgh: Office of Chief Researcher, Scottish Executive.

[82] Brandao de Souza L, Pidd M (2011). Exploring the barriers to lean health care implementation. Public Money Manag.

[83] Lukas CV, Holmes SK, Cohen AB (2007). Transformational change in health care systems: an organizational model. Health Care Manag Rev.

[84] Ben-Tovim DI, Bassham JE, Bolch D, Martin MA, Dougherty M, Szwarcbord M (2007). Lean thinking across a hospital: redesigning care at the flinders medical Centre. Aust Health Rev.

[85] Brandao de Souza L (2009). Trends and approaches in lean healthcare. Leadersh Health Serv.

