# Supplementary material for: A realist evaluation to identify contexts and mechanisms that enabled and hindered implementation and had an effect on sustainability of a lean intervention in pediatric healthcare
Source: BMC Health Serv Res. 2019 Nov 29;19:912. doi: 10.1186/s12913-019-4744-3 (PMC6884784; doi:10.1186/s12913-019-4744-3)
Supplement: Supplementary file 1 — Additional file 1. Interview Guide. [file 12913_2019_4744_MOESM1_ESM.docx]

**Interview Guide for Healthcare Providers**

**Part A. General Questions about Lean**

1. What Lean principles are you familiar with?
2. Can you tell me about your involvement in Lean? (e.g., Lean activities)
3. What Lean /tools activities are you familiar with?
4. Can you tell me how using Lean principles/ tools/ activities in your work feels for you?
5. What extent do you believe Lean to be embedded in your work/organization?

**Part B. Detailed questions about the integration of Lean**

1. How is Lean different to your previous usual ways of working?
2. How and to what extent have Lean principles/activities become a normal part of your work?
3. What are the challenges to integrating Lean into your everyday work?
4. What factors have been critical to how integrated Lean is in your work?
5. Tell me an example of when something was tried under Lean in your work setting that did become integrated? What is your understanding of why it did become integrated into practice?
6. Tell me an example of when something was tried under Lean in your work setting that didn’t become integrated? What is your understanding of why it didn’t become integrated into practice?
7. How has Lean affected the nature of your work?
8. Tell me about any benefits from using Lean that you see for your work?
9. How strongly would you say you support the use of Lean for healthcare?
10. Can you tell me your main reasons for supporting Lean or not?
11. What do you think are the core values of Lean?
12. How are Lean values congruent/incongruent with your own values?
13. How and by whom has the continued use of Lean been driven in your organization?
14. What factors unique to pediatric healthcare facilitate or hinder the continuation of Lean?

**Interview Guide for Unit Managers/Senior Leaders**

**Part A. General Questions about Lean**

1. What Lean principles are you familiar with?
2. Can you tell me about your involvement in Lean? (e.g., Lean activities)
3. What Lean activities are you familiar with?
4. Can you tell me how using Lean principles/ activities in your work feels for you?

**Part B. Detailed questions about the integration of Lean**

1. How and to what extent have Lean principles/activities become a normal part of your work/ unit/organization?
2. What are the challenges to integrating Lean into the everyday work of your unit/organization?
3. What factors have been critical to how integrated Lean is in your work/ unit/organization?
4. Tell me an example of when something was tried under Lean in your work setting that did become integrated? What is your understanding of why it did become integrated into practice?
5. Tell me an example of when something was tried under Lean in your work setting that didn’t become integrated? What is your understanding of why it didn’t become integrated into practice?
6. How has Lean affected the nature of your work/unit/organization?
7. Tell me about any benefits from using Lean that you see for your work/unit/organization?
8. How strongly would you say you support the use of Lean for healthcare?
9. Can you tell me your main reasons for supporting Lean or not?
10. What do you think are the core values of Lean?
11. How are Lean values congruent/incongruent with your own values/ teams values/ organizations values?
12. How and by whom has the continued use of Lean been driven in your organization?
13. What factors unique to pediatric healthcare facilitate or hinder the continuation of Lean?

**Interview Guide for Patient Advisory Group**

**Part A. General Questions about Lean**

1. What Lean principles are you familiar with?
2. What Lean activities are you familiar with?

**Part B. Detailed questions about the integration of Lean**

1. Tell me about your involvement in Lean?
2. Tell me about any values of Lean that you see for patients/child health?
3. How would you know a unit is using Lean? How would it be different to a unit not using Lean?
4. Do you support the use of Lean? Why?
5. Please give an example of something that has been tried under Lean?
6. What factors are important for Lean to work and to be continued?
7. How does Lean makes a difference to child healthcare?
8. How does Lean influence child health outcomes?
