# Supplementary material for: A realist evaluation to identify contexts and mechanisms that enabled and hindered implementation and had an effect on sustainability of a lean intervention in pediatric healthcare
Source: BMC Health Serv Res. 2019 Nov 29;19:912. doi: 10.1186/s12913-019-4744-3 (PMC6884784; doi:10.1186/s12913-019-4744-3)
Supplement: Supplementary file 2 — Additional file 2. CMOc visual model. [file 12913_2019_4744_MOESM2_ESM.pdf]

Additional File 2. Context, mechanism and outcome configurations: a visual model

Mechanisms (Resources & Reasoning) **Outcomes**

Frontline healthcare providers felt "push" approach — Resistance from front line healthcare providers

Senior leadership Lean training focus led by external consultancy — Leadership capacity and buy in to Lean "better equipped"

Compulsory Lean training- Didn't connect factory to patient care- feeling of disconnect for front staff — Potential reason for staff retention issues

Large amounts of Lean events- staff didn't feel involved, didn't understand the purpose — Resistance from front line healthcare providers

Contexts for early implementation

Value congruency to some degree, training failed to show the connection between Lean and healthcare — Lack of buy-in (frontline)

Mandated and funded implementation by Ministry

Perceived as an outsider that didn't fit and understand healthcare context — Lack of fit between outside culture of lean and healthcare context

External consultancy to lead implementation — Lean language did not make sense and resonate with staff, staff felt monitored

Negative media messaging — Created negative perceptions about Lean "cost cutting" — Resistance from front line healthcare providers

Hierarchical organizational structures — Leaders support Lean and value it more than frontline staff — A disconnect in the level of support & buy into Lean between senior leaders, unit managers and frontline staff

Rapidly changing contexts "innovation fatigue" — Staff overwhelmed, feel Lean is the latest fad — Lack of support for the continuation of Lean

Process of customization to local context, External consultancy JBA contract ended

***Ripple-effect:** *negative outcomes of implementation (e.g., resistance, lack of customization and negative perceptions), resources (e.g, poor training that did not connect the meaning of Lean to healthcare, external Lean consultants that were not from healthcare), and the scale of implementation (e.g., mandated top-down approach at macro level) shaped the contexts (resistance, lack of customization and negative perceptions and variation in Lean training and exposure) mechanisms (e.g., sense-making, staff engagement, awareness) and outcomes (e.g., degree of support, continuation and normalization) of Lean efforts.*

Mechanisms (Resources & Reasoning) **Outcomes**

Contexts for sustainability

Process of moving from overt Lean language & principles to implicit Lean — Continuation of Lean driven from senior leaders & unit managers

External consultancy contract ended

Degree of sense-making and staff engagement (+/-) — Degree of support, continuation and normalization of Lean in practice

Dropped use of Lean Language

Perceived positive mindset shift *(Facilitated by dropping of Lean language &external)* — Degree of support, continuation and normalization of Lean

Resistance from frontline healthcare providers

Perception that Lean is gone & was a waste of time and money, a "fashion fad" — Frontline staff are unaware of what changes are as a result of Lean or not

Negative perceptions about Lean

Unit managers/leaders feel a lot of staff are not aware of what changes are as a result of Lean or not

Unit managers recognize need for staff involvement in order for changes to stick — Frontline staff involved in Lean events and changes

Degree of observability that Lean works — Staff more likely to support and buy-in to Lean efforts for

A model to depict the interconnecting CMO configurations and ripple-effects from early-stage implementation outcomes to contexts for sustainability and their influence on the normalization and support for the sustainability of Lean
