# Supplementary material for: A realist evaluation to identify contexts and mechanisms that enabled and hindered implementation and had an effect on sustainability of a lean intervention in pediatric healthcare
Source: BMC Health Serv Res. 2019 Nov 29;19:912. doi: 10.1186/s12913-019-4744-3 (PMC6884784; doi:10.1186/s12913-019-4744-3)
Supplement: Supplementary file 3 — Additional file 3. Interview quotes to support CMOcs. [file 12913_2019_4744_MOESM3_ESM.docx]

**Additional File 3. Evidence to support CMOcs**

| **Evidence to support CMOc1** |
| --- |
| “It was externally sort of put on people, rather than asking them if there were elements of this model that were workable in their work environment. (interview 20, case 3, clinical nurse educator) |
| “I think the media has played a huge role in peoples’ opinions of what Lean is and what actually is involved with Lean. For a while there, everything that didn’t go right was, “Well pfft, friggen’ Lean.” (interview 22, case 3, unit manager**)** |
| “I personally felt talked down to by the whole thing. I felt that giving me an example would have done the same as watching how many of us fold up a paper airplane how many times and do whatever. The video, that was okay. But just the general talking to of people I just thought was not appropriate.” (interview 25, case 4, registered nurse) |
| “Well I think because it’s really, it’s hard to understand it when you’re just being taught, when you’re not really involved in the process in detail and not many of the staff have been.” (interview 5, case 1, clinical nurse educator) |

**Evidence to support CMOc 2**

| “Well I guess, it’s sometimes feels like a factory, the Lean, and when we’re trying to give more personal care, it just seems very sometimes it can feel very impersonal with Lean. So I think that’s why maybe it doesn’t work in some ways. We’re not a factory, and a lot of things are laid out like it’s supposed to be for a factory, so it doesn’t always work that way, because we have so many different situations, so many different patients, there’s so many variations whereas a factory often is the same thing over and over again.” (interview 2, case 1 , registered nurse) |
| --- |
| “It's confusing to me anyways. Lean, yes, we're pairing things down to its simplest form, but healthcare isn't simple. It's very complex and very different. Again, into the grey. Whereas on paper and people talking maybe a streamline black and white thing, healthcare is not black and white. There is a lot of situations that make it different from this patient to this patient and there's no one way that is perfect. So I don't know.” (interview 25, case 4, registered nurse) |
| “From what I know about Lean, I don’t really support it. It was a model that came from industry. Healthcare’s much more complex than industry. We’re not making widgets on an assembly line. We’re dealing with people and life and death events in this unit. They’re children. So, to try and minimize things and put it in an industrial model I think is really illogical, if not cruel.” (interview 20, case 3, clinical nurse educator). |
| “As long as there’s a room for the patient-centered, family-centered care that realizes it’s not, yeah. That’s the two to debate, whether it’s like, you’re functioning like systems, like efficiency, that kind of thing versus the family and being flexible and that kind of thing. So merging those two can be hard I guess sometimes.” (interview 3, case 1, registered nurse) |

**Evidence to support CMOc 3**

| “You know, it’s difficult for me to figure out what’s done because of Lean and what was just changed on this unit because I feel like we have so much change that I don’t necessarily know what was changed due to Lean.” (interview 19, case 1, unit manager) |
| --- |
| I think part of why people don’t buy into things is that constant change. One month we’re doing it this way. Six weeks later or six months later, well we’re not doing that anymore because we’ve run out of money or we’ve run out of momentum or the people whose job that was to roll that forward are gone or whatever. You know, so when people see that after a while it’s like how important is that? I’m not even going to listen because in six months is going to be something different.” (interview 20, case 3, clinical nurse educator) |
| “I think for me, the challenge has been trying to find a way to keep staff engaged with it, right? I think pediatric [unit name] had a lot of Lean, quality improvement work done through it, like a lot, before my time. And you know when I first started there and talked about it you could just see employee’s facts go up as they were quality improvement exhausted, like they kind of hit a wall with it.” (interview 16, case 2, unit manager) |
| “There’s a blur, so because we’re getting a new children’s hospital, there’s been a lot of change in prep for the children’s hospital, and then there’s also just in general, there’s been changes in the whole hospital, region-wide with Lean and that kind of stuff. So sometimes I’m not totally sure whether it’s hospital-related that we’re changing things, or if it’s because of Lean I guess.” (interview 2, case 1 , registered nurse) |

**Evidence to support CMOc 4**

| “Yeah. I think taking a step back too I think when we initially started down kind of a Lean process stuff as a region, we were really focusing on kind of the terms and definitions of those types of things. And I think that didn’t really resonate with staff, or some staff.” (interview 16, case 2, unit manager) |
| --- |
| “Like I honestly don’t even think about Lean anymore, I just don’t seem to hear about it much, as much as I used to.” (interview 4, case 1, care assistant) |
| “The terminology, again, staff hated the terminology and it wasn't real to them. And so, I would often change phrases to stuff that made sense to them. And like one of them was like on the Gemba. They hated that phrase. They didn't get it.” (interview 33, case 1, unit manager) |
| “We know that staff need to be involved and we're much better at just figuring out how to involve them. And I think staff are expecting now to be involved. They're expecting to be asked.” (interview 33, case 1, unit manager) |
| “Not knowing that everything we do is related to the Lean principles. And I think that’s the change. Like everything isn’t labelled Lean anymore, it’s quality improvement. So, I think taking that Lean [name of consultancy company] label away has helped with the buy in. (interview 22, case 3, unit manager) |
| “I feel like the terminology just even isn’t used as much, and so it’s not kind of in people’s minds as much I don’t think.” (interview 5, case 1, clinical nurse educator) |
| “I think it’s embedded on everyday work. And the nurses are seeing it. Whether they’re labelling it as Lean or not. (interview 22, case 3, unit manager) |

**Evidence to support CMOc 5**

| “There is a bit of congruency. I honestly want people to be cared for in the safest possible way. I want the things that people need where they need them. And of course in my position now, I mean I need things to be financially responsible decisions. Ultimately, I still have a very hard time with the fact that we’re trying to run a healthcare system like a business and we are providing a service, which lots of businesses do but we’re providing a service to people when they’re in the most fragile states they can be. So I think that we need to be very cognizant of the fact that we can’t cut corners or try to make things more efficient when people just need time and everybody is different.” (interview 19, case 1, unit manager) |
| --- |
| “It was at first difficult for me to understand why we were taking a business model that is strictly business and applying it to a service field. But the more I looked at it, I did realize that there were some benefits that we could definitely take out of that as far as Kanban and that kind of stuff. I guess less waste. It’s probably the main benefit. But I do think it’s difficult to take a business model and really apply it to a service industry. (interview 19, case 1, unit manager) |
| “I don’t know, I support for sure teamwork and principles of it, but I don’t feel like I’m fully educated on Lean itself, I just take bits and pieces maybe and don’t really realize what’s Lean and what’s not, and it’s more principles that Lean has adopted that I maybe agree with, but do I fully support Lean? Not totally sure.” (interview 3, case 1, registered nurse) |
| “I think it kind of comes back to your other question. I think it’s got to be cross-disciplinary in the sense that if you are going to institute system change it’s got to involve all the system’s players. And so maybe part of my ignorance is just a reflection of the fact that not everybody is included in sort of an understanding of what the goals are as a system.” (interview 27, case 3, physician) |
| “I think that the whole general philosophy that people have about Lean nobody would ever give credit to Lean because they’re just so – like the whole dogma is like “Oh that was such a waste of time.” But I think if you really look at it there were some good things that came from it just nobody would ever want to give credit to Lean.” (interview 29, case 2, registered nurse) |
| “Honestly the only thing I think that hinders the whole concept of Lean is bad publicity, people not understanding what it is, and that damage that happens. And I think that for staff just kind of the terminology that they didn't understand and they didn't always like having strangers in the unit with clipboards coming to measure them.” (interview 33, case 1, unit manager) |
